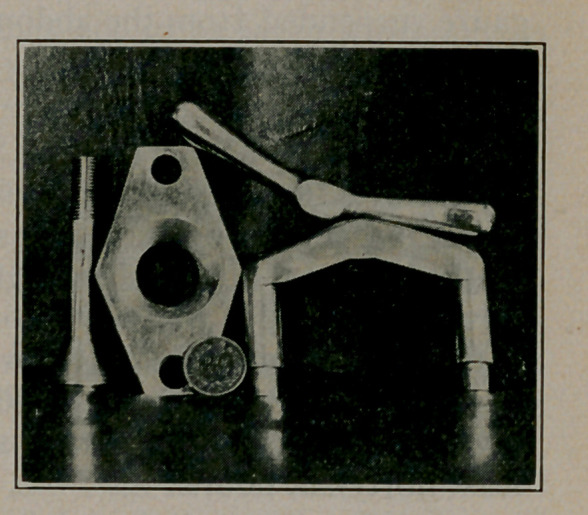# An Instrument for Circumcision

**Published:** 1914-01

**Authors:** Joseph S. Lewis

**Affiliations:** Buffalo, N. Y.


### An Instrument for Circumcision

By DR. JOSEPH S. LEWIS. Buffalo, N. Y.

THAT venerable operation circumcision, usually considered no more serious than a tonsillectomy, not infrequently proves a source of grave complications at the hands of those unskilled in operative technique, and occasionally even under the best auspices. Therefore in order to provide a method which would prove more safe and simple, especially for the physician who never undertakes a more formidable operation, the writer has devised an instrument with which circumcision may be done without danger of removing too much or too little foreskin, with minimal chance for infection, and without the need of sutures.

The following observations suggested to the writer that the foreskin might be *pinched* off instead of being cut off. A

Fig. 1.

Fig. 2.

haemostat clamped on the foreskin for a few moments leaves the clamped skin in a parchment-like condition. A bone-forceps will pinch off the epidermal part of the foreskin, leaving a closed wound with little or no bleeding; but then the wound must be opened when the epidermis is drawn back in order to trim off the mucous membrane, which can not be cut off in the same way without snipping off the glans as well.

The writer two years ago designed a tubular knife against which a second tubular knife was to cut off the foreskin after it had been drawn back over the first, previously fitted over the penis. This design a certain instrument-maker kept in his shops without progress for over a year. Meanwhile another instrument, much simpler to use as well as to make, was devised, which after numerous trials seems admirably suited to its purpose. The accompanying illustrations should require only a brief interpretation to make clear the technique.

1. The foreskin is seized by its preputial portion laterally with two mosquito clamps.

2. After crushing the dorsal and ventral portions *just past the prepuce* with a haemostat, the crushed parts are cut through, which allows the foreskin to be withdrawn in order to release adhesions and remove the smegma. If the skin be sufficiently elastic a dorsal incision alone will be necessary.

3. The *bell* of the instrument is annointed with sterile vaseline, which will allow it easily to slip in place, over glans and under foreskin. (Fig. 1.) The *platform* and *key* should also be lubricated along the parts in contact during use.

4. The bell-handle is now passed through the beveled hole in the platform, while its threaded portion is passed through the hole in the *frame*, and the key is slowly turned down drawing the circumference of the bell against the flattened edge of the beveled hole with great force. (Figs. 2 and 3.)

5. The bell-handle is grooved for a projection in the frame so that the bell may not turn while the key turns. The result is that the bell is made to bite or crush the foreskin against the platform. While the key is still tight, the foreskin is cut away from bell and handle. After waiting *five* minutes the penis is released, leaving a parchment-like ribbon a milimeter in width, all around the new union of skin and mucous membrane. This ribboned edge retracts slightly in a groove, will not bleed, and should be touched with tincture of iodine. With infants it will be best to cover the penis with vaselined gauze, to be changed with each diaper.

For various sized organs there are three sizes of bell, and three equivalent platforms, all designed to fit the same key and frame. They are measured by the size of the hole in the platform, the diameters being respectively ¾ and 1½ inches. The photograph shows the smallest platform and bell with the common frame and key, as also a copper for the sake of comparison.

Knowing of no similar instrument, the writer would name it a circumcisor. As it is not yet on the market anyone desirous of using a like instrument may have from the writer the name of the maker. There are a few points to be emphasized if the instrument is to give satisfaction.

1. Lubricate the working parts lest they jam and make it difficult to unscrew the key.

2. In slipping the bell under the foreskin fit it so that it conforms to the ellipse of the corona. The edge of the bell therefore will be oblique, not at right angles, to the shaft of the penis.

3. Turn the key down with force as far as it will go, to form the ribbon.

4. After cutting the foreskin loose, leave the key turned down tight for at least five minutes to assure permanence of the ribbon. If released too soon there is danger of late separation of the pinched edge requiring the application of sutures. Except with infants, no dressing will be needed unless it be a square flap of gauze suspended from the abdomen by a strip of adhesive plaster.

11 Irving Place.

---

Rectal Administration of Salvarsan. Rajat, *Ann. des mal ven.*, Nov., 1912, advocates he has employed this method in 125 cases and found the result as satisfactory as in the intravenous method without any of the dangers connected with the latter. The salvarsan is added to 120 c.c. of artificial serum in a solution of 5:1000. Soda may be added to increase the solubility of the salvarsan. The injection is preceded by an enema and should be given through a high rectal tube to insure its retention for at least thirty-six hours.

---

A Chemical Basis for Cancer. Dr. Howard W. Nowell, in *Boston Medical and Surgical Journal,* June 5, 1913, reports the isolation of a crystalline end product of carcinoma, highly toxic in character and of specific lethal characteristics.

Injections into rabbits produces carcinomatous lesions with metastatic foci and characteristic cachexia. Repeated injections of very small doses immunized a large number of rabbits, and serum from these animals injected into non-immune ones antagonized the toxic action of the tumor substances.

---

Male Menstruation. Dr. Guy P. Levering, Lafayette, Ind., *Ind. Med. Jour.,* Oct., 1913, reports a case in a man aged 34, half negro, a quarter Indian and a quarter French, normal except for sexual apathy and the occurrence of vesical hemorrhages every four weeks. Exploratory incision revealed no trace of internal female organs and apparently no prostate. The testicles were removed and though stated to be true male glands, the haemorrhages were relieved.

## Figures and Tables

**Fig. 1. f1:**
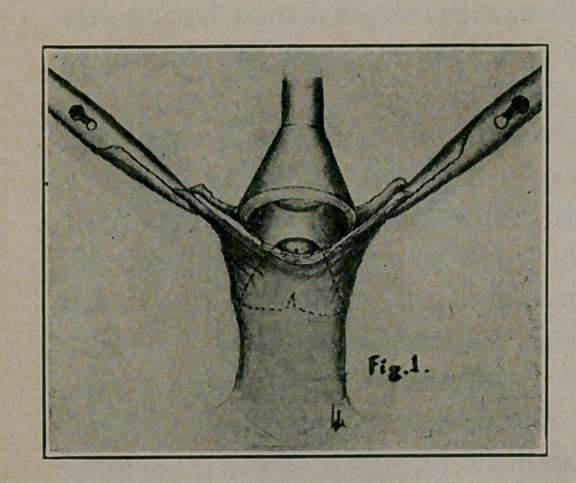


**Fig. 2. f2:**
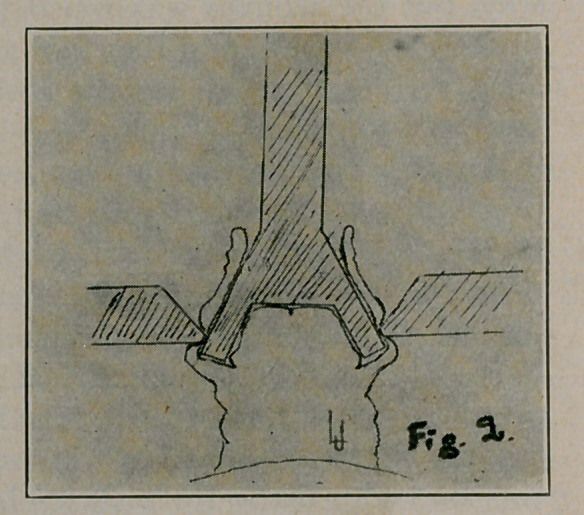


**Fig. 3. f3:**
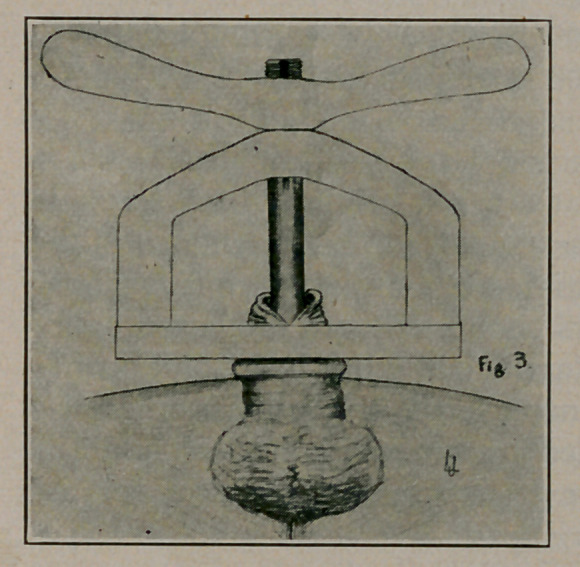


**Figure f4:**